# Cyclovirobuxine D Induced-Mitophagy through the p65/BNIP3/LC3 Axis Potentiates Its Apoptosis-Inducing Effects in Lung Cancer Cells

**DOI:** 10.3390/ijms22115820

**Published:** 2021-05-29

**Authors:** Cheng Zeng, Tingting Zou, Junyan Qu, Xu Chen, Suping Zhang, Zhenghong Lin

**Affiliations:** 1 School of Life Sciences, Chongqing University, Chongqing 401331, China; cqzengcheng@163.com (C.Z.); tingtzou1997@163.com (T.Z.); qujunyan1229@yeah.net (J.Q.); chenxu_2021@163.com (X.C.); 2 Shenzhen Key Laboratory of Precision Medicine for Hematological Malignancies, Department of Pharmacology, Base for International Science and Technology Cooperation: Carson Cancer Stem Cell Vaccines R&D Center, International Cancer Center, Shenzhen University Health Science Center, Shenzhen 518055, China

**Keywords:** cyclovirobuxine D, BNIP3, mitophagy, apoptosis, lung cancer

## Abstract

Mitophagy plays a pro-survival or pro-death role that is cellular-context- and stress-condition-dependent. In this study, we revealed that cyclovirobuxine D (CVB-D), a natural compound derived from *Buxus microphylla*, was able to provoke mitophagy in lung cancer cells. CVB-D-induced mitophagy potentiates apoptosis by promoting mitochondrial dysfunction. Mechanistically, CVB-D initiates mitophagy by enhancing the expression of the mitophagy receptor BNIP3 and strengthening its interaction with LC3 to provoke mitophagy. Our results further showed that p65, a transcriptional suppressor of BNIP3, is downregulated upon CVB-D treatment. The ectopic expression of p65 inhibits BNIP3 expression, while its knockdown significantly abolishes its transcriptional repression on BNIP3 upon CVB-D treatment. Importantly, nude mice bearing subcutaneous xenograft tumors presented retarded growth upon CVB-D treatment. Overall, we demonstrated that CVB-D treatment can provoke mitophagy and further revealed that the p65/BNIP3/LC3 axis is one potential mechanism involved in CVB-D-induced mitophagy in lung cancer cells, thus providing an effective antitumor therapeutic strategy for the treatment of lung cancer patients

## 1. Introduction

Lung cancer is one of the most common malignant tumors and a frequent cause of death worldwide [1,2]. Surgical resection and radical radiotherapy can offer patients a high probability of a cure at the presentation stage of lung cancer. Unfortunately, the majority of patients diagnosed with lung cancer are at an advanced stage of their disease, leading to a relatively poor outcome [3,4]. The combined treatment of etoposide or irinotecan with platinum for lung cancer therapy is the standard first-line method, although some cytotoxic agents such as paclitaxel, docetaxel, gemcitabine and vinorelbine were used in phase II clinical trials with modest efficacy. To date, very few drugs are approved as effective candidates for lung-cancer therapy [5]. Therefore, it is very urgent to develop new therapeutic agents for the treatment of lung cancer patients.

Cyclovirobuxine D (CVB-D), a natural bioactive alkaloid component derived from *Buxus microphylla*, is primarily used to treat cardiovascular and cerebrovascular disease [6,7]. Recently, mounting evidence indicates that CVB-D may possess antitumor effects in various kinds of cancers [8,9,10,11]. However, the underlying mechanism remains to be investigated.

Autophagy is defined as an evolutionally conserved process of recycling excess intracellular macromolecules, which are engulfed in autophagosomes, subsequently degraded in autolysosomes and ultimately, are broken down into their constituent parts [12,13]. Autophagy is known to be a pro-survival stress response through which cells can maintain their own energy homeostasis under certain energy-deficiency conditions by removing aggregated proteins and damaged organelles [14]. Mitophagy is a kind of well-documented mitochondrial quality-control system that mediates the clearance of dysfunction or damaged mitochondria through the selective autophagy of mitochondria. Two pathways have been well identified to mediate mitophagy initiation in mammalian cells including parkin-Pink1. Parkin is an E3 ubiquitin ligase recruited by Pink1, which translocates to mitochondria to mediate mitophagy initiation [15]. Both BNIP3 (BCL2 and adenovirus E1B 19-kDa-interacting protein 3) and BNIP3L (also known as NIX) are localized to mitochondria and are atypical BH3-only members of the BCL2 family [16]. BNIP3 was first identified in a yeast two-hybrid screen as BCL2 and adenovirus E1B-19 K-interacting protein. The transcription of BNIP3 can be activated by transcription factor HIF1α [17] and FOXO3a [18] and transcriptionally silenced by p65 [19,20,21]. BNIP3 and BNIP3L have been reported to participate in hypoxia-induced mitophagy [22]. Mechanistically, BNIP3 interacts with microtubule-associated protein LC3 to form a mitochondria-BNIP3-LC3-autophagosome complex and mediates mitophagy initiation [23,24].

Under certain conditions, the occurrence of mitophagy is of great importance for cells to preserve a population of normal-function mitochondria and protect cells from cellular damage [25,26]. There have been reports that mitophagy can not only protect cells from death but also demonstrates pro-death functions in response to various chemicals. It is perceived that mitophagy may play dual roles in response to anticancer treatment, depending on the property of chemical substances. Therefore, the dissection of the underlying role of mitophagy involved in cancer therapy is crucial for improving anticancer efficiency through the pro-survival or pro-death role of mitophagy.

In this study, we demonstrated that CVB-D-induced lung-cancer cell death is closely associated with mitophagy activation. CVB-D treatment causes significant depletion of mitochondria, which is accompanied by the upregulation of the mitophagy receptors BNIP3 and BNIP3L. Our results further demonstrated that the upregulation of BNIP3 is due to the downregulation of p65, which relieves its inhibition on BNIP3 expression. Notably, mitophagy induced by CVB-D leads to a further increase of apoptotic death in lung cancer cells. These findings provide a cross link between mitophagy and apoptosis upon CVB-D treatment, which indicates that CVB-D is a potential candidate and is of particular clinical relevance for lung-cancer treatment.

## 2. Results

### 2.1. CVB-D Induces Cell Cycle Arrest and Cell Death

**Figure 1:**
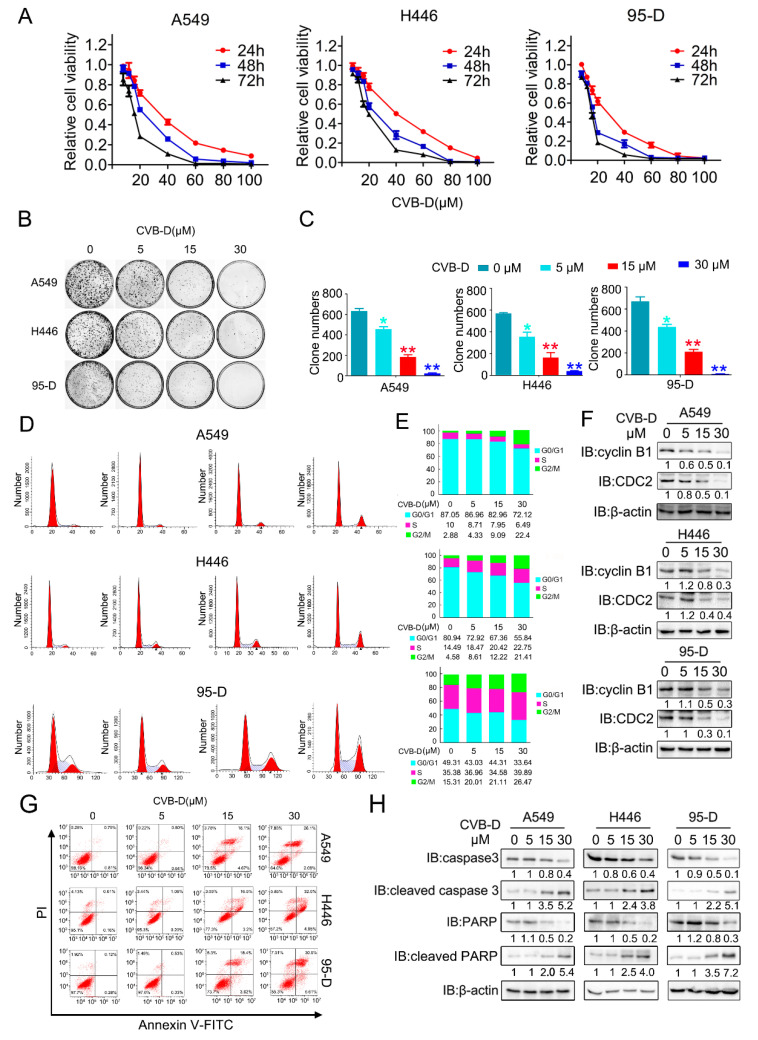
CVB-D inhibits the growth and promotes the apoptosis of lung cancer cells. (**A**) Cell counting kit-8 (CCK8) assay of A549, H446 and 95-D upon CVB-D treatment was performed at the indicated time and concentration. (**B**,**C**) A colony formation assay was performed in lung cancer cells through the treatment with CVB-D with the indicated concentration for 14 days (**B**). Representative images (left) and the quantification of the colony number (right) are shown (**C**). Error bars represent data from three independent experiments (mean ± SD). (* *p* < 0.05, ** *p* < 0.01). (**D**,**E**) Flow cytometric analysis of cell cycle in A549, H446 and 95-D cells upon CVB-D treatment was performed at the indicated concentration for 24 h (**D**). A statistical analysis of cell-cycle distribution is shown (**E**). (**F**) The expression of CDC2 and cylinB1 in lung cancer cells upon CVB-D treatment at the indicated concentration for 24 h was detected through an immunoblotting analysis. Beta-actin was used as the loading control. (**G**) Flow cytometric analysis of apoptosis in A549, H446 and 95-D cells upon CVB-D treatment was performed at the indicated concentration for 24 h. (**H**) The expression of caspase3, cleaved caspase3, PARP and cleaved PARP in A549, H446 and 95-D cells upon CVB-D treatment at the indicated concentration for 24 h was detected through an immunoblotting analysis. Beta-actin was used as the loading control.

To evaluate whether CVB-D exhibits antitumor effects against lung cancer cells, the CCK8 (cell counting kit-8) assay was performed to evaluate the cytotoxic effects of CVB-D against lung cancer cells (A549, H446 and 95-D). As shown in Figure 1A, incubation with CVB-D resulted in reduced cell viability in all cell lines in a time- and dose-dependent manner. Consistent with the results of the CCK8 assay, CVB-D also dramatically decreased the colony numbers in all lung cancer cells in a dose-dependent manner (Figure 1B,C). Together, the above results suggest that CVB-D has a considerable antitumor effect in killing lung cancer cells in vitro.

Cell cycle regulation plays a key role in antitumor therapy, and we therefore tested whether CVB-D treatment can induce cell-cycle arrest in lung cancer cells. As expected, exposure to CVB-D treatment caused significant G2/M arrest in all lung cancer cells (Figure 1D,E). Consistent with previous reports that CDC2 (CDK1) and cyclinB1 (CCNB1) are crucial to G2/M transition [27,28], CVB-D-induced G2/M arrest was confirmed by the decrease in G2/M-transition-related protein CDC2 and cyclin B1 in a dose-dependent manner in all lung cancer cells (Figure 1F). To test whether the cytotoxic effects of CVB-D leads to cell death, we then measured apoptosis by examining the apoptotic ratio of lung-cancer cell lines upon CVB-D treatment with PI and FITC-annexin V double staining. Flow cytometric analysis revealed that CVB-D treatment induced an obvious apoptotic phenotype in lung cancer cells in a dose-dependent manner (Figure 1G), which can be further confirmed by the increased cleavage of caspase 3 and PARP under CVB-D treatment (Figure 1H). Taken together, these data indicated that the cytotoxic effects of CVB-D against lung cancer cells are due to cell-cycle arrest and apoptosis induction.

### 2.2. Transcriptional Analysis Identified That Mitochondria Event May Be a Potential Contributor to the CVB-D-Induced Cell Death

**Figure 2:**
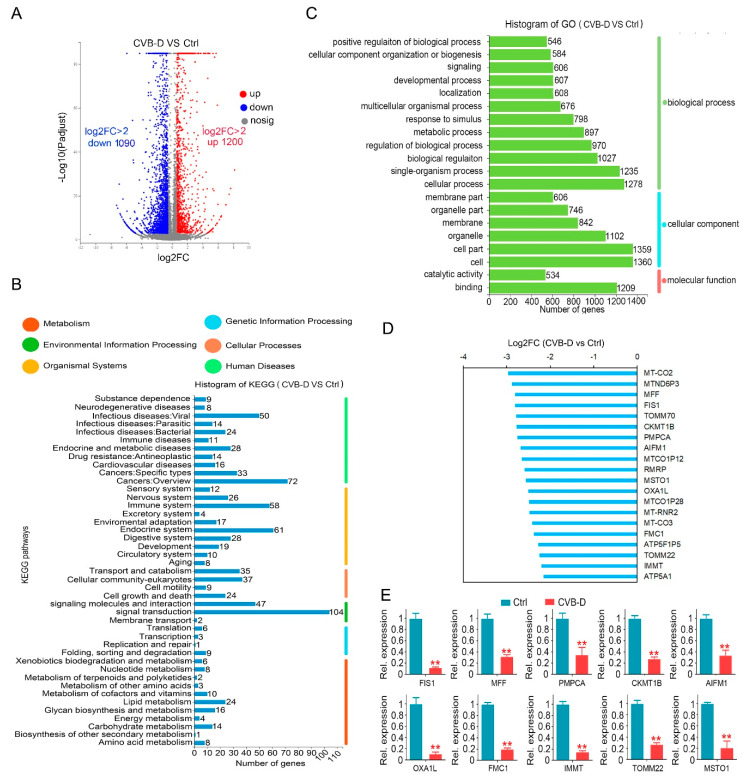
The downregulation of mitochondria-related genes contributes to CVB-D-induced cell death. (**A**) A volcano plot shows the number of upregulated and downregulated genes upon 30 μM CVB-D treatment for 24 h. (**B**) KEGG classification of DEGs (differentially expressed genes) in A549 cells upon 30 μM CVB-D treatment for 24 h. (**C**) GO function classification of DEGs in A549 cells upon 30 μM CVB-D treatment for 24 h. (**D**) Top 20 downregulated mitochondria-related genes are shown upon 30 μM CVB-D treatment for 24 h. (**E**) Downregulated mitochondria-related genes were validated by using qRT-PCR upon 30 μM CVB-D treatment for 24 h. Error bars represent data from three independent experiments (mean ± SD) (** *p* < 0.01).

RNA-seq was applied to investigate the underlying mechanism of apoptotic induction upon CVB-D treatment. Of these differentially expressed genes (DEGs) upon 30 μM CVB-D treatment, 1200 genes were upregulated (log2FC>2), and 1090 genes were downregulated (log2FC>2) (Figure 2A). For functional pathway analysis, these DEGs are involved in 43 pathways and are mostly enriched in signaling transduction, indicating their involvement in the regulation of cell viability and apoptosis (Figure 2B). For function annotation in the GO database, the DEGs were mostly enriched in the following biological processes (BP) terms: “single-organism process” and “cellular process”, the cellular component (CC) terms “cell” and “cell part” and the molecular function (MF) terms “binding” (Figure 2C). Interestingly, we found various mitochondria-related genes were downregulated upon CVB-D treatment (Supplementary Table S1), and the top 20 downregulated mitochondria-related genes are shown (Figure 2D), which indicated that a mitochondria event may be involved in CVB-D induced cell death. Next, we conducted qRT-PCR to confirm the expression changes in mitochondria-related genes (Figure 2E).

### 2.3. CVB-D Causes the Dysfunction and Loss of Mitochondria in Lung Cancer Cells

**Figure 3:**
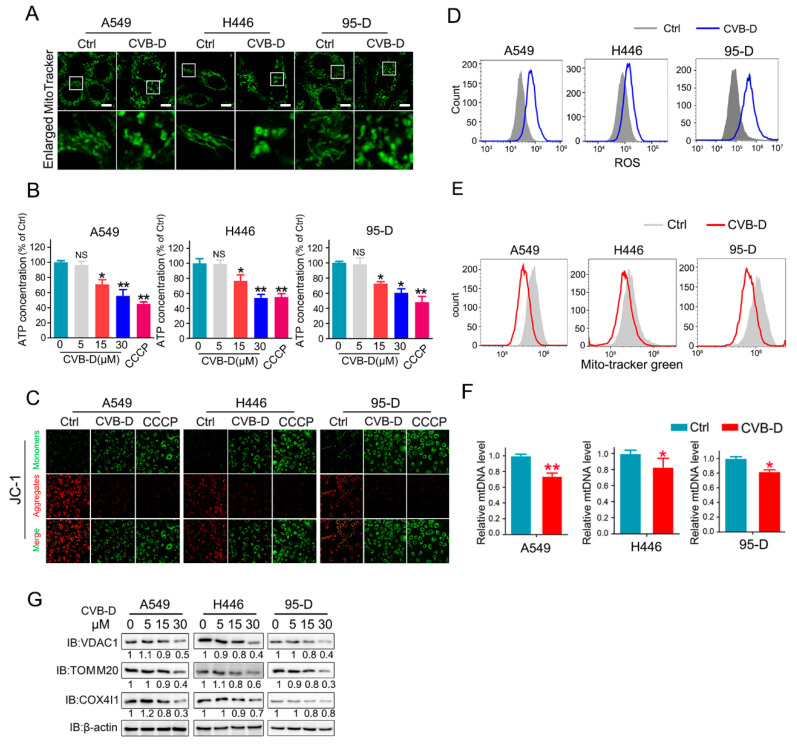
CVB-D induces mitochondria depletion in lung cancer cells. (**A**) Images of mitochondrial morphology in lung cancer cells treated with or without 30 μM CVB-D for 24 h are shown, (scale bar, 10 µM). (**B**) Analysis of the ATP level upon CVB-D treatment in A549, H446, and 95-D cells at the indicated concentration for 24 h. CCCP was used as a positive control (20 μM, 12 h) (NS *p* > 0.05, * *p* < 0.05, ** *p* < 0.01). (**C**) A JC-1 probe assay was performed to examine the mitochondrial membrane potential changes in A549, H446 and 95-D cells treated with or without 30 μM CVB-D for 24 h. CCCP was used as a positive control (20 μM, 6 h). (**D**) Flow cytometric analysis was performed to detect the ROS level of A549, H446 and 95-D cells with or without 30 μM CVB-D treatment for 24 h. (**E**) Flow cytometric analysis was performed to detect the mitochondrial mass by mito-tracker green staining with or without 30 μM CVB-D treatment for 24 h. (**F**) qRT-PCR analysis was performed to test the DNA copy number of mitochondria in A549, H446 and 95-D cells treated with or without 30 μM CVB-D treatment for 24 h. Error bars represent data from three independent experiments (mean ± SD) (* *p* < 0.05, ** *p* < 0.01). (**G**) The expression of VDAC1, TOM20 and COX4I1 proteins in A549, H446 and 95-D cells upon CVB-D treatment at the indicated concentration for 24 h was detected by an immunoblotting analysis.

Transcriptome data showed that CVB-D treatment resulted in the downregulation of various mitochondria-related genes in A549 cells, which implied that mitochondria might be a potential factor involved in CVB-D-induced apoptosis. To test our hypothesis, we first evaluated the morphology change of mitochondria upon CVB-D treatment. In the control group, large number of normal mitochondria with tubular-shaped morphology can be seen in lung cancer cells, while CVB-D treatment led to a significant morphology change of mitochondria into an abnormal structure (Figure 3A), suggesting that the normal function of mitochondria may be disturbed upon exposure to CVB-D treatment. Alongside the abnormal structure of mitochondria upon CVB-D treatment, we detected a decreased ATP (adenosine triphosphate) level in all lung cancer cells. CCCP (carbonylcyanide-m-chlorophenylhydrazone) was used as a positive control to decrease ATP production (Figure 3B), indicating that CVB-D treatment causes the dysfunction of mitochondria. Moreover, we detected a decrease of MMP (mitochondrial membrane potential) in lung cancer cells (CCCP was used as a positive control to monitor the change of MMP) (Figure 3C). Next, we measured cellular reactive oxygen species generation to further address mitochondrial functional alterations. Compared with the control group, CVB-D treatment resulted in a significant increase in ROS level in lung cancer cells (Figure 3D). Considering the effects of CVB-D in disturbing the normal function of mitochondria, we next aimed to detect the change of mitochondria quantity upon CVB-D treatment. Compared with the control group, CVB-D treatment caused a significant decrease in mitochondrial mass, as measured by staining the mito-tracker green (Figure 3E). Consistent with the decreased mitochondrial mass, exposure to CVB-D also led to a significant reduction in mitochondrial DNA and mitochondrial proteins, including VDAC1 (voltage dependent anion channel 1), TOMM20 (translocase of outer mitochondrial membrane20) and COX4I1 (cytochrome c oxidase subunit 4I1) (Figure 3F,G). Together, these results indicated that CVB-D treatment caused mitochondria dysfunction and mitochondria loss.

### 2.4. CVB-D Treatment Induces Mitophagy

**Figure 4:**
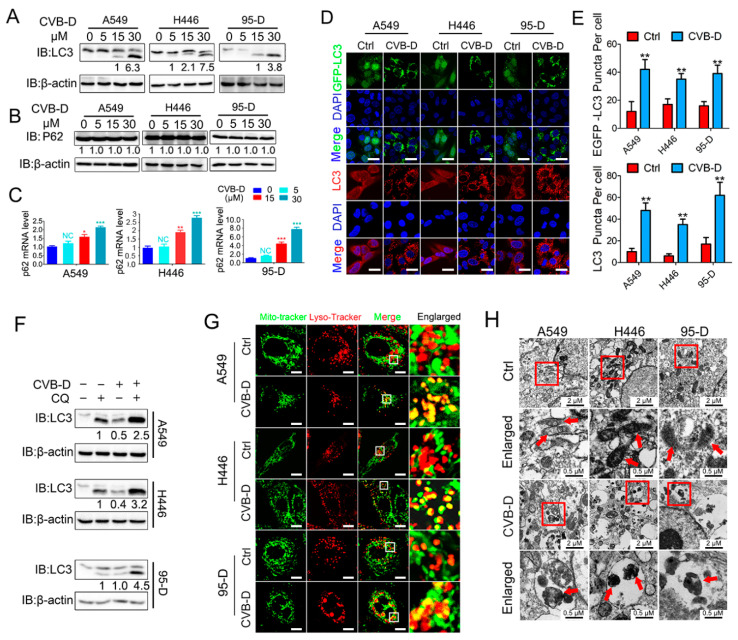
CVB-D induces mitophagy in lung cancer cells. (**A**,**B**) An immunoblotting analysis of LC3 or SQSTM1/p62 expression in A549, H446 and 95-D cells upon CVB-D treatment at the indicated concentration for 24 h. (**C**) qRT-PCR analysis was performed to detect the expression of p62 in A549 and 95-D cells upon CVB-D treatment at the indicated concentration for 24 h. Error bars represent data from three independent experiments (mean ± SD) (* *p* < 0.05, ** *p* < 0.01, *** *p* < 0.001). (**D**,**E**) Immunofluorescence analysis of GFP-LC3 and endogenous LC3 in A549, H446 and 95-D cells treated with or without 30 μM CVB-D for 24 h (scale bar, 20 µM) (**D**) and the quantification of puncta is shown (**E**). Error bars represent data from five different horizons (mean ± SD) (** *p* < 0.01). (**F**) An immunoblotting analysis of LC3 expression in A549, H446 and 95-D cells upon treatment with CVB-D (30 μM) or CQ (10 μM) alone or together for 24 h. (**G**) A confocal microscope was used to detect the colocalization of mitochondria and lysosomes stained with mito-tracker green or lyso-tracker red in A549, H446 and 95-D cells treated with or without 30 μM CVB-D for 24 h (scale bar, 10 µM). (**H**) The induction of mitophagy was detected with a transmission electron microscope upon CVB-D treatment at the indicated concentration for 24 h. A red arrow indicates mitochondria.

Mitophagy is an important mitochondria quality-control pathway that removes dysfunctional or damaged mitochondria to maintain normal cell growth. Given that CVB-D treatment causes mitochondrial dysfunction and decline, we then aimed to test whether CVB-D treatment provokes mitophagy. LC3I conversion to LC3II, which is regarded as a molecular marker of autophagy, was analyzed by Western blot. As expected, CVB-D treatment led to a significant conversion of LC3I to lipidated LC3II in a dose-dependent manner in all tested cells (Figure 4A). SQSTM1/p62 is a kind of autophagy adaptor that binds ubiquitin and LC3 to participate in the autophagy process. Its downregulation in the autophagy process was defined as a hallmark of autophagy processing [29]. Next, we tested the expression level of SQSTM1/p62 upon CVB-D treatment. To our surprise, the protein level of selective cargo receptor SQSTM1/p62 was not visibly influenced by CVB-D treatment (Figure 4B), but qRT-PCR results showed that the mRNA of p62 was significantly elevated (Figure 4C). This suggested that the degradation of the SQSTM1/p62 protein in autophagy was counterbalanced by the increase in SQSTM1/p62 translation upon CVB-D treatment. CVB-D treatment also increased exogenous GFP-LC3 and endogenous LC3 puncta in all lung cancer cells, suggesting the formation of autophagosomes (Figure 4D,E). The formation of puncta in cells generally correlates with either the induction of autophagy or the blockage of autophagosome–lysosome formation in the late stage of autophagy. Therefore, we next combined treatment with CVB-D and the autolysosome inhibitor chloroquine (for the inhibition of late-stage autophagy) to further confirm the initiation of autophagy upon CVB-D treatment. As shown in Figure 4F, combined treatment with CVB-D and chloroquine (CQ) further increases the conversion of LC3I to LC3II, suggesting that CVB-D treatment induces complete autophagy in lung cancer cells. Additionally, CVB-D treatment significantly increased the colocalization of mitochondria and lysosomes, as evidenced by the merged fluorescent signaling of mitotracker-green and lysotracker-red (Figure 4G), which implied the activation of mitophagy. The activation of CVB-D-induced mitophagy was also confirmed through transmission electron microscopy, which showed that mitochondria were surrounded by autolysosomes, an indication of the formation of mitolysosomes in A549, H446 and 95-D cells (Figure 4H). Taken together, these results showed that CVB-D induced mitophagy in lung cancer cells.

### 2.5. CVB-D Induces Mitochondrial Dysfunction and Loss by Provoking Mitophagy

**Figure 5:**
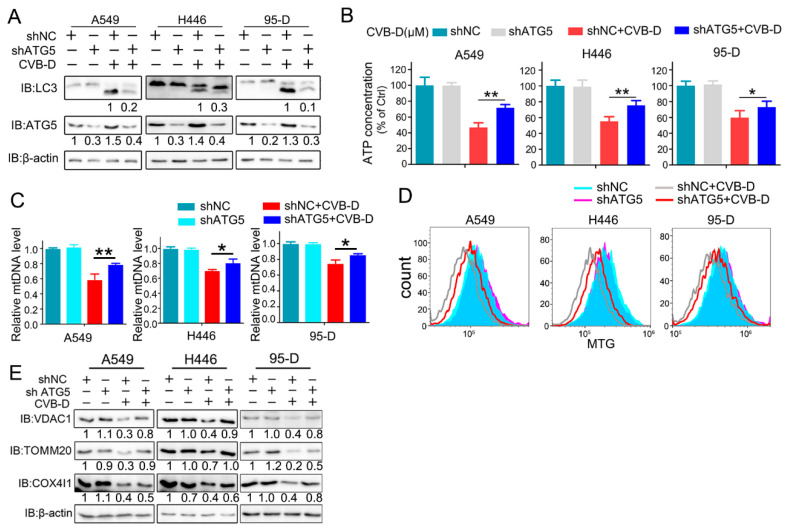
Mitophagy inhibition partially restores mitochondria dysfunction and loss. (**A**) Control and ATG5 knockdown lung cancer cells were treated with or without 30 μM CVB-D for 24 h, and LC3 expression was detected through a Western blot analysis. (**B**) The relative ATP levels in ATG5 knockdown and control lung cancer cells treated with or without 30 μM CVB-D. Error bars represent data from three independent experiments (mean ± SD) (** *p* < 0.01, * *p* < 0.05). (**C**) A qRT-PCR analysis of the mitochondrial DNA copy number in control and ATG5 knockdown lung cancer cells treated with or without 30 μM CVB-D. Error bars represent data from three independent experiments (mean ± SD) (** *p* < 0.01, * *p* < 0.05). (**D**,**E**) Flow cytometric analysis of mitochondrial mass (**D**) and an immunoblotting analysis of VDAC1, TOM20 and COX4I1 expression in the control and ATG5 knockdown cells treated with or without 30 μM CVB-D for 24 h is shown (**E**).

We next evaluated whether mitochondrial dysfunction and loss is ascribed to mitophagy activation upon CVB-D treatment. A shRNA against ATG5 (an essential component in the autophagy process, conjugating with ATG12 to participate in the formation of autophagosomes) was applied to explore the underlying mechanism in CVB-D-induced mitochondrial loss. As expected, the knockdown of ATG5 significantly inhibited the conversion of LC3I to lipidated LC3II upon CVB-D treatment in all lung cancer cells (Figure 5A). Importantly, the knockdown of ATG5 can significantly rescue CVB-D-induced mitochondria dysfunction, as evidenced by a relatively lower decrease in ATP level in ATG5 knockdown cells compared with the shNC group upon CVB-D treatment (Figure 5B), indicating that CVB-D-induced mitophagy causes mitochondria dysfunction. Additionally, ATG5 knockdown significantly antagonized the reduction of mitochondrial DNA upon CVB-D treatment (Figure 5C). Consistent with the above, the reduced mitochondrial mass was also significantly rescued in ATG5-knockdown cells, compared with the control, in response to CVB-D treatment (Figure 5D). In addition, the expression of mitochondrial proteins (VDAC1, TOMM20 and COX4I1) was restored in ATG5 knockdown cells (Figure 5E). Collectively, these data indicated that CVB-D induced mitochondria dysfunction and loss is partially attributed to mitophagy activation.

### 2.6. Inhibition of Mitophagy Attenuates Apoptosis

**Figure 6:**
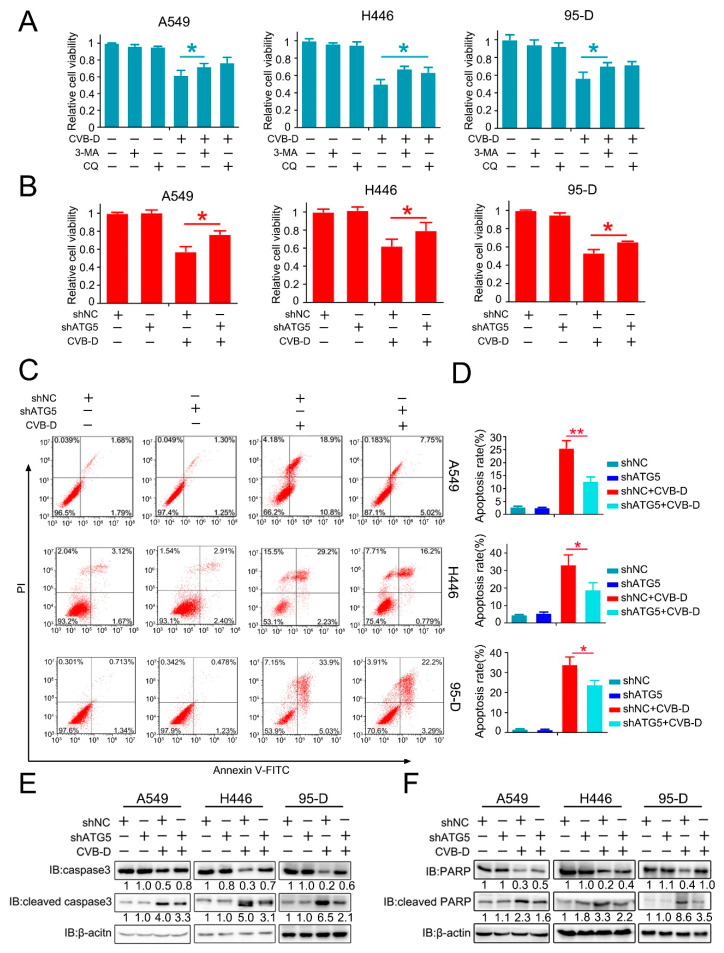
Mitophagy inhibition partially attenuates the cell death of lung cancer cells. (**A**) A cell counting kit-8 (CCK8) assay was performed in lung cancer cells treated with CVB-D, alone or together with 3-MA (5 mM) or CQ (20 μM). 3-MA and CQ were pretreated for 2 h and the medium was replaced with 30 μM of CVB-D (without 3-MA or CQ) for 24 h. Error bars represent data from three independent experiments (mean ± SD) (* *p* < 0.05). (**B**) A cell counting kit-8 (CCK8) assay was performed on the control and ATG5 knockdown lung cancer cells treated with or without 30 μM CVB-D for 24 h. Error bars represent data from three independent experiments (mean ± SD) (* *p* < 0.05). (**C**,**D**) Flow cytometric analysis was performed to detect the apoptosis of shATG5 or shNC lung cancer cells treated with or without 30 μM CVB-D for 24 h. Error bars represent data from three independent experiments (mean ± SD) (** *p* < 0.01, * *p* < 0.05). (**E**,**F**) The expression of caspase 3, cleaved caspase 3, PARP and cleaved PARP in shATG5 or shNC lung cancer cells treated with or without 30 μM CVB-D for 24 h was detected by immunoblotting.

The role of CVB-D-induced mitophagy in disturbing the normal function of mitophagy in lung cancer cells and mitochondria is vitally important as an energy supplement to maintain the normal growth of cells; therefore, we presumed that this kind of mitophagy induced by CVB-D may contribute to cell apoptosis. Next, we aimed to investigate whether mitophagy induced by CVB-D was engaged in CVB-D-induced apoptotic death. Thus, the pharmacological and genetic inhibition of autophagy was applied to clarify the underlying connection between mitophagy and cytotoxicity upon CVB-D treatment. As expected, combined treatment with CVB-D and autophagy inhibitor (CQ or 3-methyladenine, 3-MA) resulted in an alleviation of cytotoxicity in all lung cancer cells (Figure 6A), and a similar result was obtained from ATG5 knockdown cells (Figure 6B), indicating that the activation of mitophagy induced by CVB-D partially reinforces cytotoxicity. The same result was obtained in ATG5 knockdown cells, suggesting that CVB-D reduced apoptosis in ATG5 knockdown cells compared with the control group, as tested by flow cytometry with PI and FITC-annexin V double staining (Figure 6C,D). Additionally, consistent with the above results, the knockdown of ATG5 also decreased the expression of cleaved caspase 3 (Figure 6E) and cleaved PARP (Figure 6F). Thus, these results implied that mitophagy-mediated mitochondrial dysfunction upon CVB-D treatment at least partially serves as a crucial event to provoke the apoptosis of lung cancer cells.

### 2.7. CVB-D Triggers Mitophagy through p65/BNIP3/LC3 Axis

**Figure 7:**
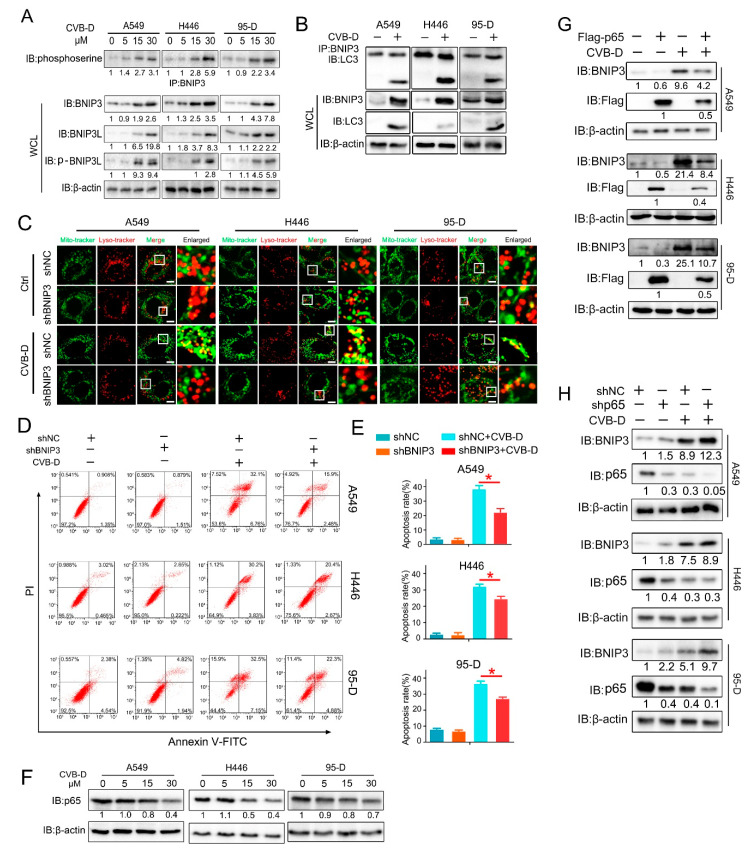
CVB-D triggers mitophagy through the p65/BNIP3/LC3 pathway in lung cancer cells. (**A**) Immunoblotting analysis showed the expression of BNIP3 and BNIP3L in A549, H446 and 95-D cells treated with CVB-D at the indicated concentration for 24 h. (**B**) Coimmunoprecipitation and immunoblotting analysis were performed to detect the interaction of BNIP3 and LC3 in lung cancer cells upon 30 μM CVB-D treatment for 24 h. (**C**) The colocalization of mitochondria and lysosomes in shBNIP3 and shNC lung cancer cells was detected by confocal microscope (scale bar, 10 µM) upon 30 μM CVB-D treatment for 24 h. (**D**,**E**) Flow cytometric analysis was performed to detect the apoptosis of shBNIP3 or shNC lung cancer cells treated with or without 30 μM CVB-D treatment for 24 h. Error bars represent data from three independent experiments (mean ± SD) (* *p* < 0.05). (**F**) Immunoblotting analysis of p65 expression in A549, H446 and 95-D cells upon CVB-D treatment at the indicated concentration for 24 h. (**G**,**H**) Immunoblotting analysis of BNIP3 expression in p65 overexpression (**G**) and knockdown (**H**) lung cancer cells with or without 30 μM CVB-D treatment for 24 h.

The parkin-Pink1 and BNIP3/BNIP3L pathways are known to mediate mitophagy activation in mammalian cells. To further explore the underlying mechanism involved in CVB-D-induced mitophagy, we first detected the expression of parkin/Pink1 and BNIP3/BNIP3L in lung cancer cells upon CVB-D treatment. The results showed that parkin exhibits decreased expression in all lung cancer cells, and Pink1 showed a decreased expression in 95-D cells, but no obvious change was detected in A549 and H446 cells (Supplementary Figure S1). We concluded that the parkin-Pink1 pathway may be not the major contributor involved in CVB-D-induced mitophagy. Next, we tested the expression of BNIP3 and BNIP3L to see whether their expression was affected by CVB-D treatment. As shown in Figure 7A, we detected a significant elevation of BNIP3 and BNIP3L expression in lung cancer cells in a dose-dependent manner upon CVB-D treatment. We also detected an increased phosphorylation level in both BNIP3 and BNIP3L, which is consistent with previous reports that the phosphorylation modification of BNIP3 and BNIP3L promote mitophagy [30,31]. It implied that the BNIP3/BNIP3L pathway may be involved in CVB-D-induced mitophagy and that it mediates mitophagy initiation. Previous studies showed that BNIP3 interacted with microtubule-associated protein LC3 to form a mitochondria-BNIP3-LC3-autophagosome complex and that it mediates mitophagy activation. Therefore, we examined whether CVB-D treatment can enhance the interaction of BNIP3 with LC3. Coimmunoprecipitation and Western blot analysis showed that CVB-D treatment significantly increased their interaction (Figure 7B). We next aimed to investigate whether the upregulation of BNIP3 is a key factor in mediated mitophagy activation upon CVB-D treatment. shRNA against BNIP3 was applied to test whether the knockdown of BNIP3 can weaken CVB-D-induced mitophagy. As expected, the knockdown of BNIP3 significantly inhibited the activation of CVB-D-induced mitophagy as visualized by the decrease of the colocalization of mitochondria and lysosomes (Figure 7C and Supplementary Figure S2). Additionally, BNIP3 knockdown significantly decreases CVB-D-induced apoptotic death compared with control cells (Figure 7D,E).

BNIP3 can be transcriptionally activated by HIF1α or FOXO3a or transcriptionally silenced by p65 [17,18,19,20]. CVB-D treatment failed to promote the expression of HIF1α and FOXO3a (Supplementary Figure S3). Notably, p65 was downregulated by CVB-D treatment in all lung cancer cells (Figure 7F). To explore whether p65 inhibition is a potential factor in mediating BNIP3 upregulation in CVB-D-treated lung cancer cells, we established p65 overexpression and knockdown cells to explore its roles in CVB-D-induced BNIP3 upregulation. The results showed that the overexpression of p65 significantly weakens CVB-D-induced BNIP3 expression (Figure 7G) while the knockdown of p65 significantly enhances it (Figure 7H). Altogether, these results suggest that CVB-D induced mitophagy in lung cancer cells by targeting the p65/BNIP3/LC3 pathway.

### 2.8. CVB-D Inhibits Tumor Growth

**Figure 8:**
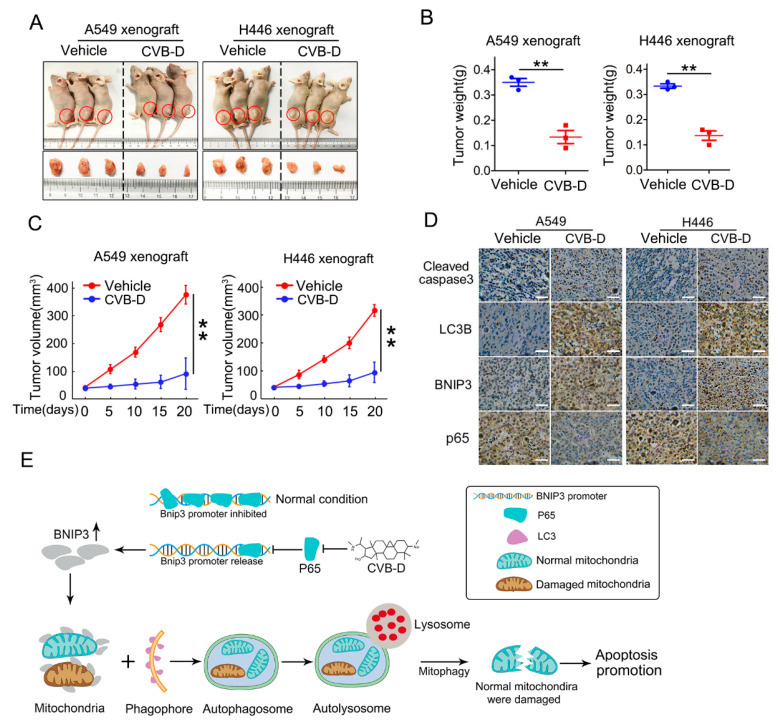
CVB-D inhibits tumor growth in vivo. (**A**–**C**) Representative photos showing euthanized mice and xenograft tumors (**A**) at four weeks postsubcutaneous injection (*n* = 3 per group). Tumor sizes were measured every five days. Tumor weight (**B**) and tumor volume (**C**) are shown. Error bars represent data from three independent experiments (mean ± SD) (** *p* < 0.01). (**D**) Immunohistochemical staining of cleaved-caspase 3, LC3B, BNIP3 and p65 in A549 and H446 xenograft mice treated with or without CVB-D (scale bar, 200 µM). (**E**) A schematic model showing the function of upregulation of BNIP3 induced by CVB-D in mitophagy induction and apoptosis.

To evaluate the antitumor effects of CVB-D in vivo, nude mice bearing subcutaneous A549 and H446 xenografts were established to evaluate the antitumor ability of CVB-D. As shown in Figure 8A, in both A549 and H446 xenograft nude mice, CVB-D dramatically repressed tumor growth as quantified by tumor weight (Figure 8B) and tumor volume (Figure 8C). In line with the above results, we detected elevated expression of cleaved caspase3, LC3B and BNIP3 and decreased expression of p65 in xenograft tissues from the CVB-D treatment group, compared with the vehicle (Figure 8D). Taken together, these data indicated that CVB-D-induced mitophagy serves as a contributing factor in the apoptotic death of lung cancer cells upon CVB-D treatment, and the working model is shown in Figure 8E.

## 3. Discussion

In this study, we revealed that CVB-D, a natural compound derived from *Buxus microphylla*, can provoke apoptotic death in lung cancer cells. In addition to apoptotic death, CVB-D treatment can also significantly provoke mitophagy activation. Under certain conditions, mitophagy serves a protective role by removing dysfunction or damaged mitochondria to alleviate cellular stress. The extent and duration of mitophagy activation is a crucial impact factor for cell survival or death decisions, so we conducted a series of experiments to address the role of CVB-D-induced mitophagy in the apoptosis of lung cancer cells. Interestingly, we found that mitophagy induced by CVB-D serves a pro-death role in CVB-D-treated lung cancer cells by promoting mitophagy-mediated mitochondrial dysfunction. It is apparent that mitophagy induced by CVB-D is more likely to be overactivated and that this kind of mitophagy serves as a destroyer of mitochondria in lung cancer cells and ultimately potentiates CVB-D-induced apoptotic death. Our results further demonstrated that the p65/BNIP3/LC3 axis is a potential mediator for mitophagy activation upon CVB-D treatment. Treatment of lung cancer cells with CVB-D can significantly suppress the expression of p65, a transcriptional suppressor of BNIP3, and the downregulation of p65 can significantly relieve its inhibition on BNIP3 transcription upon CVB-D treatment and can cause enhanced expression of BNIP3, thus enhancing its interaction with LC3 and mediating mitophagy activation. We demonstrated that CVB-D exhibits a kind of favorable anti-lung-cancer activity in vitro and in vivo and revealed a novel antitumor mechanism of CVB-D in lung cancer cells, namely that mitophagy-mediated mitochondrial dysfunction is a contributor to CVB-D-induced apoptotic death through the targeting of the p65/BNIP3/LC3 axis.

In our preliminary experiments, we found that CVB-D exhibited favorable anti-lung-cancer potential both in vitro and in vivo. Next, we investigated the underlying mechanism by which CVB-D is involved in lung-cancer cell death. A RNA-seq analysis was conducted to detect the change in gene expression upon CVB-D treatment, and we found that numerous mitochondria-related genes were significantly downregulated, so it revealed that CVB-D treatment causes direct or indirect damage to mitochondria in lung cancer cells. In line with RNA-seq results, we also provided novel evidence that CVB-D treatment induces mitochondria dysfunction and loss by testing cellular ATP concentration, mitochondrial mass and mitochondrial DNA copy-numbers, as well as the degradation of mitochondrial proteins. Along with these findings, we also revealed that mitophagy was significantly activated upon CVB-D treatment. Subsequently a series of ATG5 knockdown experiments were conducted to confirm our results. These observations strongly supported the idea that CVB-D treatment promotes mitophagy activation and that mitophagy is a contributing factor involved in mitochondria loss in lung cancer cells.

Although mitophagy is a specific autophagy that selectively removes dysfunctional or damaged mitochondria to protect cells from death [32], abnormal or overactivated mitophagy is toxic to cells. Our observation revealed that CVB-D-induced mitophagy was seemingly overactivated, as evidenced by the knockdown of ATG5 significantly relieving the CVB-D-induced mitochondrial ATP reduction, which is similar to previous reports that mitochondrial dysfunction mediated by mitophagy is a switch for cell death fate decisions [33,34]. Indeed, these conclusions can be verified by the observations that the genetic abolishing of the mitophagy process can significantly alleviate cell apoptosis upon CVB-D treatment; therefore, CVB-D-induced mitophagy contributes to apoptosis activation. Based on the above, we concluded that CVB-D treatment causes the overactivation of mitophagy and that this kind of mitophagy contributes to cell apoptosis induction by causing dysfunction and damage to healthy mitochondria.

Notably, we found that mitophagy induced by CVB-D is not associated with the parkin-Pink1 pathway, a well-studied pathway involved in mitophagy activation. CVB-D treatment caused a significant decrease in parkin protein in all lung cancer cell lines upon CVB-D treatment in a dose-dependent manner for 24 h, suggesting that CVB-D-induced mitophagy in lung cancer cells is independent of parkin, at least at our time point. A previous study showed that chemical-induced mitophagy is not accompanied by the enhanced expression of parkin but with the enhanced expression of Pink1 recruited to outer membrane mitochondria [35]. Next we examined whether Pink1 is involved in CVB-D-induced mitophagy. In our time point, CVB-D treatment caused a significant decrease in Pink1 expression in H446 and 95-D cells but no visible change in A549. These differential expressions upon CVB-D treatment in lung cancer cells may depend on the cellular content. Given this, we concluded that Pink1 is also not involved in CVB-D-induced mitophagy. But for a short time of CVB-D stimulation (within 24 h), the manner of change in the expressions of parkin and Pink1 needs further clarification. P62 is a kind of adaptor protein. In this study, we detected no significant p62 protein expression change, but a significantly enhanced expression of p62 mRNA was seen, suggesting that the degradation of the SQSTM1/p62 protein in autophagy was counterbalanced by the increase in SQSTM1/p62 translation upon CVB-D treatment, but whether p62 was involved in CVB-D induced mitophagy is also largely unknown and needs to be further clarified. In sharp contrast to the expression of parkin and Pink1, we detected a strong increase in the expression of the mitophagy receptor BNIP3. Based on this, we hypothesized that the BNIP3 receptor may be a major candidate to mediate mitophagy activation upon CVB-D treatment, and further evidences confirmed our hypothesis that the reduced expression of BNIP3 by knocking down significantly abolished the CVB-D-induced colocalization of mitochondria and lysosomes. BNIP3-mediated mitophagy is closely related to the interaction of BNIP3 and LC3 (23). In analogy to previous findings, our results revealed that CVB-D treatment induced the upregulation of BNIP3 expression and resulted in tighter interaction of BNIP3 and LC3. Altogether, based on the above results, we concluded that CVB-D-induced mitophagy is associated with the enhanced expression of the BNIP3 receptor, which mediated the activation of mitophagy. Several studies showed that BNIP3 and BNIP3L could cause the opening of the mitochondrial permeability transition pore (mPTP). Therefore, the overexpression of BNIP3 and BNIP3L contributes to the collapse of MMP (mitochondrial membrane potential) (16,22). In this study, CVB-D may also function as a destroyer by directly causing mitochondrial damage by enhancing the expression of BNIP3. These intricate relationships between BNIP3-mediated apoptosis-promoting mitophagy and the BNIP3-mediated collapse of MMP deserves further exploration. BNIP3 can be transcriptionally activated by several transcription factors, including HIF1α and FOXO3a. Unexpectedly, these regulators are not involved in the CVB-D-induced upregulation of the BNIP3 protein and our data further showed that the downregulation of p65 (BNIP3 transcriptional suppressor) was closely associated with CVB-D-induced BNIP3 upregulation.

In physiological conditions, mitochondrial quality and mass are accurately controlled by undergoing mitochondrial biogenesis and mitophagy constantly [26]. Generally, mitophagy is a kind of survival strategy for cells using the degradation of dysfunctional or damaged mitochondria. In certain physiological conditions, for example, in the process of ischemic preconditioning, the activation of mitophagy could significantly mediate the degradation of damaged mitochondria and protect kidneys from damage [36]. In oxidative stress conditions, the activation of mitophagy is also of great importance to maintain bone-marrow mesenchymal stem-cell survival [37]. In screening potential antitumor drugs, many chemicals were also found to provoke mitophagy. Usually, the kind of mitophagy induced by chemicals serves a protective role for cancer cells to alleviate cytotoxicity, for example, retigeric acid B-induced mitophagy can significantly antagonize cell death, and the inhibition of mitophagy can improve the chemotherapeutic effects against prostate cancer cells [38]. In the ^125^I seeds brachytherapy process, ^125^I seeds irradiation causes both mitochondrial damage and mitophagy activation, and the activated mitophagy contributes to the clearance of damaged mitochondria to maintain cellular homeostasis and survival [39]. Contrary to the above results, our data demonstrated that CVB-D-induced mitophagy serves a pro-death role in lung cancer cells by causing mitochondrial dysfunction, thus promoting apoptosis activation. Previous reports demonstrated that ceramide targets autophagosomes to mitochondria, causing lethal mitophagy [40] and that mitophagy contributes to pulmonary epithelial cells’ necroptosis in response to cigarette smoke exposure [41]. BAY 87-2243 (inhibitor of complex I (CI) of the mitochondrial respiratory chain)-induced Pink1-dependent mitophagy is responsible for melanoma cell death [42]. Ketoconazole-induced mitophagy is a crucial contributor to provoking apoptosis [34]. Similar to previous reports, our results demonstrated that mitophagy induced by chemicals exhibits cytotoxicity to cells (abnormal or excessive mitophagy). Although the pro-survival or pro-death role of mitophagy induced by chemicals depends on the property of the chemicals, there remains controversy about the roles of chemicals-induced autophagy/mitophagy in cell death and survival, and it seems that it is cellular-content-dependent. For example, AT 101 triggers mitophagic cell death in glioma cells [33] but triggers protective autophagy in MCF7 cells [43].

Based on our findings that mitophagy induced by CVB-D could significantly potentiate the apoptotic death of lung cancer cells, it is certain that mitophagy induced by CVB-D is excessive and toxic to lung cancer cells. Despite our experimental evidence proving that CVB-D induced mitophagy and promoted apoptotic cell death, the knockdown of ATG5 or BNIP3 led to a partial alleviation of cytotoxicity. Therefore, it is undeniable that other factors may be involved in CVB-D-triggered cell death and need to be further elucidated.

In this study, we showed that CVB-D demonstrated favorable anti-lung-cancer activity both in vitro and in vivo and that it has potential application in the treatment of lung cancer. Based on its autophagy/mitophagy induction activity, its potential application in the treatment of other diseases also deserves to be explored. Under certain conditions, the activation of autophagy may serve as a treatment strategy to defend against intracellular bacteria, parasites and virus infection by targeting intracellular microorganisms (xenophagy) and mediating the clearance of microorganisms or via modulating the antiviral immune response of organisms to defend against infection [44]. In terms of anti-infection, whether CVB-D possesses potential applications is largely unknown and deserves to be explored.

## 4. Materials and Methods

### 4.1. Cell Culture, Reagents and Antibodies

Human lung cancer cell lines (A549, H446 and 95-D) were purchased from the Cell Bank of the Chinese Academy of Sciences (Shanghai, China), and cultured in DMEM containing 10% fetal bovine serum (FBS) and a 100 U/mL mixture of penicillin and streptomycin. All cells were kept at 37 °C and 5% CO2. Cyclovirobuxine D (CVB-D) was purchased from the Chengdu Alpha Biotechnology Incorporation and dissolved in methanol to generate a 70 mM stock solution. The primary antibodies for Western blotting, immunoprecipitation and immunohistochemistry against target proteins are as follows: caspase3 (Zen Bioscience, 300968, Chengdu, China), cleaved-caspase3 (Zen Bioscience, 380169), PARP (Zen Bioscience, 380451), cleaved-PARP (Zen Bioscience, 380374), VDAC1 (Sangon Biotech, D124100, Shanghai, China), TOMM20 (Sangon Biotech, D153158), COX4I1 (Sangon Biotech, D262690), LC3 (Sangon Biotech, D163557), ATG5 (Sangon Biotech, D121650), SQSTM1/p62 (Beyotime, AF5312), BNIP3 (Zen Bioscience, 381756), BNIP3L (Zen Bioscience, 381891), β-actin (Santa Cruz, sc-47778), p65 (Cell Signaling Technology, 8242S, Danvers, MA, USA), CDC2 (Cell Signaling Technology, 9116S), cyclinB1 (Cell Signaling Technology, 4135S), p-BNIP3L (phosphoS81) (abcam, ab208190, Cambridge, UK) and phosphoserine (Millipore, AB1603, Burlington, MA, USA).

### 4.2. Cell Viability and Colony Formation Assay

Cell viability was assessed through a cell counting kit-8 (CCK8) assay (MedChemExpress, HY-K0301, Monmouth Junction, NJ, USA). Briefly, cells were seeded in 96-well plates at a concentration of 5 × 103 per well, incubated overnight, then subjected to different concentrations of CVB-D treatments and cultured for 24, 48 or 72 h. The CVB-D-contained medium was replaced with 100 μL fresh DMEM; CCK8 (10 μL/well) was added, and then incubated at 37 °C for 2 h, and the absorbance value was measured at a 450 nm wavelength (BioTek, Synergy/HTX, Winooski, VT, USA). For clonogenic assay, 1 × 10^3^ cells were seeded in 35 mm cell culture dishes, cultured overnight, and then cells were pretreated with various concentrations of CVB-D for 6 h, followed by replacing the CVB-D-containing medium with a fresh medium to form colonies in 2–3 weeks. Colonies were fixed with glutaraldehyde (6.0% *v/v*) and stained with crystal violet (0.5% *w/v*) (Beyotime, C0121, Shanghai, China).

### 4.3. Flow Cytometric Analysis

A549, H446 and 95-D cells were seeded into 6-well plates, cultured overnight, and then the cells were treated with CVB-D for 24 h. Next, cells were harvested and stained with an Annexin V-FITC/PI apoptosis detection kit (CWBIO, CW2574, Beijing, China) to perform a apoptosis assay or stained with 100 nM mitotracker green (Beyotime, C1048) to perform a mitochondria mass assay. Reactive oxygen species (ROS) levels were assessed by staining with 10 μM fluorescent dye 2′7′-dichlorfluorescein-diacetate (DCFH-DA) (Beyotime, S0033S) for 20 min. For a cell-cycle analysis, cells were incubated with various concentrations of CVB-D for 24 h, rinsed in precooled PBS, followed by fixing with 75% alcohol overnight at 4 °C. Then cells were digested with 1% RNase A (Beyotime, ST579) and stained with 1mg/mL propidium iodide (PI) (Beyotime, ST511) for 30 min. All experiments were performed according to the corresponding manufacturer’s instructions. At least 10,000 live cells were collected and analyzed on the flow cytometer (Beckman, CytoFLEX, Brea, CA, USA). Experimental data were analyzed using Flow Jo 7.6 and Modifit software.

### 4.4. Immunoprecipitation and Western Blotting Analysis

For the interaction analysis of BNIP3 and LC3, lung cancer cells were treated with or without 30 μM CVB-D for 24 h, rinsed in precooled PBS (2.7 mM KCl, 137 mM NaCl, 2 mM KH2PO4 and 10 mM Na2HPO4), and total proteins were extracted using a RIPA lysis buffer (150 mM NaCl, 50 mM Tris-HCl, pH 8, 0.5% sodium deoxycholate, 1% NP-40, 0.2% SDS) containing a protease inhibitor cocktail (EDTA-free) (MedChemExpress, HY-K0011, Monmouth Junction, NJ, USA). After centrifugation (13,000× *g*, 10 min), total proteins were quantified with a BCA Protein Assay Kit (CWBIO, CW0014, Shanghai, China), and 30 μL of protein A-agarose beads (Santa Cruz, SC-2001) and 2 μg BNIP3 primary antibody were added into equal amounts of lysates and then subjected to immunoprecipitation assay overnight at 4 °C. Subsequently, the precipitates were centrifuged (1000 r/min, 3 min), resuspended and washed with washing buffer, and this process was repeated three times. Then immune complexes were eluted with SDS-PAGE loading buffer (5×, 250 mM Tris-HCL (pH 6.8), 50% (*w/v*) glycerol, 5% (*v/v*), β-mercaptoethanol, 10% (*w/v*) SDS, 0.5% (*w/v*) bromophenol blue) at 100 °C for 10 min and subjected to sodium dodecyl sulfate-polyacrylamide gel electrophoresis (SDS-PAGE) analysis. Subsequently it was transferred onto 0.45 μm PVDF membranes (GE Healthcare, A29280264) and blocked with 5% (*w/v*) skimmed-milk dissolved in a TBST buffer (150 mM NaCl, 0.05% (*v/v*) Tween 20, 20 mM Tris-HCL) for 1 h and then incubated with the corresponding primary antibodies overnight at 4 °C. The PVDF membranes were washed three times (10 min/time) with the TBST buffer. Secondary HRP-labeled goat anti-Mouse lgG (H+L) (Beyotime, A0216) or HRP-labeled goat anti-Rabbit lgG (H+L) antibodies were applied (Beyotime, A0208) at 1:5000 for 1 h at room temperature, then washed three times (10 min/time) with the TBST buffer. The immunoreactions were visualized with the Western BrightTM ECL reagent (Advansta, 191026-11, San Jose, CA, USA) using ChemiDocTM XRS+ (Bio Rad, Hercules, CA, USA). For the analysis of protein expression upon CVB-D treatment, cells were rinsed in precooled PBS, and total proteins were extracted using a RIPA lysis buffer containing protease inhibitors. After centrifugation (13,000× *g*, 10min), total proteins were quantified with a BCA Protein Assay Kit (CWBIO, CW0014, Jiangsu, China). Equal amounts of protein samples (30–80 μg) were subjected to SDS-PAGE analysis. For analysis of the phosphorylation level of BNIP3, briefly, lung cancer cells were treated with the indicated CVB-D concentration for 24 h, and total proteins were extracted using a RIPA lysis buffer containing a protease inhibitor cocktail and phosphorylase inhibitor (Sangon Biotech, C500019). After centrifugation, total proteins were quantified, and 30 μL of protein A-agarose beads and 2 μg BNIP3 primary antibody were added into equal amounts of lysates and then subjected to immunoprecipitation assay overnight at 4 °C, and the subsequent steps were conducted as in the above description. The phosphorylation modification of BNIP3 was detected using a phosphoserine antibody.

### 4.5. Cellular ATP Detection

Cellular ATP concentration was detected using an ATP Assay Kit (Beyotime, S0026B) according to the corresponding manufacturer’s standard instructions. Briefly, cells were seeded into 60 mm dishes, cultured overnight, and then cells were treated with CVB-D for 24 h. Total proteins were extracted using an ATP detection lysis buffer and quantified with a BCA Protein Assay Kit (CWBIO, CW0014). A total of 100 μg protein for each sample was subjected to the detection of ATP concentration, and chemiluminescence intensity was detected using a multi-mode ELISA reader (BioTek, Synergy/HTX, Winooski, VT, USA). ATP concentration was calculated according to the standard curve, and the relative ATP level for each sample was normalized to the control group. 

### 4.6. Plasmids and Transfection

The full length of LC3 cDNA was cloned into pEGFP-C1 vector (Addgene Company, USA) to generate a recombinant pEGFP-LC3 plasmid. To generate knockdown cell lines, shRNA against ATG5, BNIP3 and P65 was constructed into the PLKO.1 vector. Lentivirus particles were produced using a three-plasmid packaging system. pMD2G, psPAX2 and pLKO.1 (Addgene Company, Watertown, MA, USA); the 293T cells and the supernatant containing lentivirus particles were collected after transfection for 48 h. A549, H446 and 95-D cells were infected with lentivirus in the presence of 8 μg/mL polybrene (MedChemExpress, HY-112735) for 24 h and were selected with 2 μg/mL puromycin (Solarbio, IP1280, Beijing, China) for two weeks. Stable expression clones were validated by Western blotting and used in the following experiments. The target sequences were synthesized by the Sangon company (Shanghai, China) and are as follows: ATG5, forward: CCGGCCTGAACAGAATCATCCTTAACTCGAGTTAAGGATGATTCTGTTCAGGTTTTT, reverse: AATTAAAAACCTGAACAGAATCATCCTTAA CTCGAGTTAAGGATGATTCTGTTCAGG. BNIP3: forward: CCGGGCCTCGGTTTCTATTTATAATCTCGAGATTATAAATAGAAACCGAGGCTTTTT, reverse: AATTAAAAAGCCTCGGTTTCTATTTATAATCTCGAGATTATAAATAGAAACCGAGGC. P65 forward: CCGGCGGATTGAGGAGAAACGTAAACTCGAGTTTACGTTTCTCCTCAATCCGTTTTT, reverse: AATTAAAAACGGATTGAGGAGAAACGTAAACTCGAGTTTACGTTTCTCCTCAATCCG. To generate P65 overexpression cell lines, the full length of p65 cDNA was inserted into pCMV-Flag vector (Addgene Company) and transfected into A549, H446 and 95-D cells, then selected with 500 μg/mL G418 (Solarbio, IG0010) to generate p65 stable expression cell lines.

### 4.7. Determination of Gene Expression by qPCR

Total RNA was extracted using the trizol reagent (Takara, 9108, Kyoto, Japan); a total of 1 µg RNA for each sample was reverse-transcribed using SuperRT cDNA Synthesis Kit (CWBIO, CW0741), and quantitative real-time PCR was performed using the SYBR-Green qPCR master mix (CWBIO, CW0659). The qRT-PCR primers in this study were synthesized by the Sangon company (Shanghai, China) and used as follows: p62, forward: CCAGAGAGTTCCAGCACAGA, reverse: TGAGGAACAGATGGAGTCGG, β-actin, forward: TGACGTGGACATCCGCAAAG, reverse: CTGGAAGGTGGACAGCGAGG, FIS1 forward: AGCGGGATTACGTCTTCTACC, reverse: CATGCCCACGAGTCCATCTTT, MFF forward: ACTGAAGGCATTAGTCAGCGA, reverse: TCCTGCTACAACAATCCTCTCC, CKMT1B forward: CTGGTTGGACGCTAGATCAGT, reverse: GGTCAGCAAATACCTCATAGGTC, PMPCA forward: CTCCTGCGTACAGACGGTTTA, reverse: CGAAGCCCATTATCCAATGTGG, AIFM1 forward: CCCAATGCTATTGTGCAATCCG, reverse: GAAGCCACCAAAATCTGAGTCTA, OXA1L forward: GCCTCAGTACCTCTGCTATCT, reverse: TCCCACTGGGGTGTATGACC, FMC1 forward: TCCCCGTCGCACACTTTTC, reverse: TGAAGCTCATGTTGGGCTCTG, IMMT forward: CGATTCAGTCGGGTCCACTAA, reverse: AGCTGGAGTATCTCCCTTTTGT, TOMM22 forward: GGCCGGAGCCACTTTTGAT, reverse: AGGTCCTAGAAGTATCTGCCG, MSTO1 forward: ATCCCAAGAACCCTTATCTCCA, reverse: GGGAGTGGTGAGGAACCTTT. The relative gene level was normalized to β-actin using the 2^−ΔΔCT^ method. For mitochondrial DNA quantitation, total DNA was isolated with a DNA Isolation kit (CWBIO, CW0565), followed by a qPCR analysis using the mtDNA primers (synthesized by Sangon company, Shanghai, China): MT-ND1 (mitochondrially encoded NADH: ubiquinone oxidoreductase core subunit 1), forward: CCCTAAAACCCGCCACATCT, reverse: GAGCGATGGTGAGAGCTAAGGT. Nuclear target gene LPL (lipoprotein lipase), forward: ATGAGAGTTGGGTGCCAAAAC, reverse: AGTCCACCACAATGACATTGG. All experiments were performed with the standard amplification protocol according to the corresponding manufacturer’s instructions. Relative mitochondria DNA copy numbers were normalized to LPL using the 2^−ΔΔCT^ method.

### 4.8. Confocal Microscopy Imaging

Cells were cultured in confocal dishes (Biosharp, BS-15-GJM, Hefei, China) overnight and treated with 30 μM CVB-D for 24 h. The morphology of mitochondria was determined by staining 100 nM Mito-Tracker green (Beyotime, C1048) for 30 min at 37 °C. Cells were washed twice, and images were visualized under confocal microscopy (Leica TCS SP8 microscope, Wetzlar, Germany). To evaluate autophagic flux, GFP-LC3-transfected cells were treated with 30 μM CVB-D for 24 h, followed by fixation with 4% paraformaldehyde, then cells were washed twice and visualized under confocal microscopy (Leica TCS SP8 microscope). To detect the endogenous LC3, cells were treated with 30 μM CVB-D for 24 h, fixed with 4% paraformaldehyde, permeabilized with 0.2% Triton X-100, and then this process was followed by blocking with 10% goat serum. Then cells were incubated with the anti-LC3 antibody and Alexa Fluor 555 goat anti-mouse IgG (H+L) antibody, and images were visualized under confocal microscopy (Leica TCS SP8 microscope). To evaluate the colocalization of mitochondria and lysosomes, cells were treated with 30 μM CVB-D for 24 h, labeled with 100 nM Mito-Tracker green (Beyotime, C1048) and 50 nM Lyso-Tracker Red (Beyotime, C1046) for 30 min at 37 °C and visualized under confocal microscopy (Leica TCS SP8 microscope) immediately. To evaluate the change of MMP (mitochondrial membrane potential), we treated cells with 30 μM CVB-D for 24 h. Then cells were labeled with a JC-1 probe using the mitochondrial membrane potential assay kit with JC-1 (Beyotime, C2005) according to the corresponding manufacturer’s instructions, incubated for 20 min at 37 °C, washed twice and visualized under confocal microscopy (Leica TCS SP8 microscope) immediately.

### 4.9. Transmission Electron Microscopy

Briefly, cells were treated with 30 μM CVB-D for 24 h, fixed with 2.5% glutaraldehyde buffered in a 0.1 mM phosphate buffer (pH 7.4) overnight at 4 °C, washed with phosphate buffer, followed by being embedded in 1% osmium tetroxide and dehydrated in a series concentration of ethanol. Next, cell pellets were embedded in epon resin; ultrathin sections were cut and stained with 2% uranyl acetate, and images were captured using TEM (JEM-1400plus).

### 4.10. RNA Extraction, RNA-Seq Library Preparation and Sequencing

A549 cells were treated with 30 μM CVB-D for 24 h. Total RNA was extracted using the TRIZOL reagent (Takara, Kyoto, Japan), and genome DNA was digested with DNase I (Takara). The RNA library was constructed by using a TruSeqTM RNA sample preparation kit (Illumina), according to the corresponding manufacturer’s instructions, using 1 μg of total RNA. Briefly, mRNA was isolated, fragmented, and double-stranded cDNA was synthesized with random hexamer primers (Illumina, San Diego, CA, USA) using a SuperScript double-stranded cDNA synthesis kit (Invitrogen, Carlsbad, CA, USA). Subsequently, the synthesized cDNA was subjected to phosphorylation, end-repair and ‘A’ base addition according to Illumina’s library construction instructions. cDNA target fragments of 300 bp were selected and amplified. The library was subjected to sequencing with the Illumina NovaSeq 6000 sequencer (2 × 150 bp read length).

### 4.11. Immunohistochemistry Analysis

Tumors obtained from A549 and H446 xenograft mice were fixed in 4% paraformaldehyde, then were paraffin-embedded and sectioned. Tumor slides were incubated with primary antibodies at 4 °C overnight, incubated with a secondary antibody, and then the chromogenic reaction was conducted with 3, 3-diaminobenzidine (Solarbio, DA1015) and counterstained with hematoxylin (Solarbio, G1080, Beijing China) according to the corresponding manufacturer’s instructions. Images were captured using the Nikon microscope.

### 4.12. Animal Study

All animal experiments were approved by the institutional animal care and use committee of Chongqing University. Briefly, 5 × 10^6^ of A549 and H446 cells were suspended in 100 μL of PBS and subcutaneously implanted into the flank of nude mice (male, 6 weeks). When the tumor volume reached ~50 mm^3^, mice were randomly divided into different groups and were intraperitoneally injected with CVB-D (20 mg/kg) or a vehicle every two days. Tumor volume was measured every five days. The mice were sacrificed after 20 days of drug treatment, and tumors were harvested and analyzed.

### 4.13. Statistical Analysis

The Student’s t test and a one-way ANOVA were used to compare the differences of two groups or multiple groups. Data were presented as means ± SD of three independent experiments. A value of *p* < 0.05 was considered statistically significant.

## 5. Conclusions

In summary, we propose that treatment with CVB-D in lung cancer cells can provoke mitophagy through the p65/BNIP3/LC3 axis, and that this overactivated mitophagy is one of the contributing factors in apoptosis induction. Our results provided a novel insight into the mechanism of the antitumor activity of CVB-D, and its favorable anti-lung-cancer activity in vitro and in vivo indicated that CVB-D is a potential therapeutic candidate in lung-cancer treatment.

## Data Availability

The data that support the findings of this study are available from the corresponding author upon reasonable request.

## Supplementary Materials

Supplementary materials can be found at https://www.mdpi.com/article/10.3390/ijms22115820/s1.
