# Supplementary material for: Cyclovirobuxine D Induced-Mitophagy through the p65/BNIP3/LC3 Axis Potentiates Its Apoptosis-Inducing Effects in Lung Cancer Cells
Source: Int J Mol Sci. 2021 May 29;22(11):5820. doi: 10.3390/ijms22115820 (PMC8199090; doi:10.3390/ijms22115820)
Supplement: Supplementary file 1 [file ijms-22-05820-s001.zip › ijms-1228661-supplementary.pdf]

Table S1. Down-regulated mitochondria-related genes (CVB-D vs Ctrl)

	Gene	Log2FC(CVB-D/Ctrl)	P-value	significant	regulate
1	MT-CO2	-2.963754763	0.0000514	yes	down
2	MTND6P3	-2.882140997	1.42E-56	yes	down
3	MFF	-2.811751669	0.000182861	yes	down
4	FIS1	-2.811751669	0.000182861	yes	down
5	TOMM70	-2.784784622	1.67E-10	yes	down
6	CKMT1B	-2.768287479	6.8E-19	yes	down
7	PMPCA	-2.750351125	0.000029	yes	down
8	AIFM1	-2.683761747	1.48E-41	yes	down
9	MTCO1P12	-2.648252937	1.36E-13	yes	down
10	RMRP	-2.585643832	9.28E-45	yes	down
11	MSTO1	-2.561314244	0	yes	down
12	OXA1L	-2.499807663	0.00000832	yes	down
13	MTCO1P28	-2.485545454	1.45E-15	yes	down
14	MT-RNR2	-2.479211316	2.86E-41	yes	down
15	MT-CO3	-2.41121374	9.92E-09	yes	down
16	FMC1	-2.373630557	0.00000775	yes	down
17	ATP5F1P5	-2.271183288	0.00000699	yes	down
18	TOMM22	-2.247599356	2.63E-12	yes	down
19	IMMT	-2.19641552	7.15E-32	yes	down
20	ATP5A1	-2.150553583	3.61E-66	yes	down
21	ATPAF1	-2.138732992	1.99E-19	yes	down
22	MTCO3P12	-2.113826491	0.00000351	yes	down
23	ALDH2	-2.111311951	0.000000222	yes	down
24	LARS2	-2.077133946	4.16E-50	yes	down
25	MT-ND4	-2.036716963	2.53E-21	yes	down
26	MT-ND6	-2.015341433	1.43E-60	yes	down
27	VARS2	-1.959359649	1.84E-98	yes	down
28	CKMT1A	-1.949255193	0.0000454	yes	down
29	ATP5G1	-1.880233701	2.96E-166	yes	down
30	MTCO2P12	-1.870379399	2.7E-72	yes	down
31	MTND4P20	-1.835999216	7.19E-09	yes	down
32	MTG1	-1.766785403	4.3E-11	yes	down
33	ATP5B	-1.686220787	0.000222072	yes	down
34	MTCO3P39	-1.641826668	0.0000664	yes	down
35	MGME1	-1.596900797	4.62E-13	yes	down
36	MRPL50	-1.584341173	0.0000828	yes	down
37	MTCO1P22	-1.547776465	1.11E-31	yes	down
38	MRPS31P5	-1.534110274	0.00013835	yes	down
39	MCCD1P1	-1.48089045	7.54E-50	yes	down
40	NADK2	-1.443413109	1.21E-31	yes	down
41	MRPS14	-1.419434247	1.41E-55	yes	down
42	MPC1	-1.37598399	1.61E-25	yes	down
43	MTFR1L	-1.363267841	2.96E-11	yes	down
44	MRPL38	-1.342960908	2.48E-12	yes	down
45	MT-TS1	-1.323300145	0.000029	yes	down
46	MT-ATP6	-1.267927863	2.4E-16	yes	down
47	MRPL11	-1.255358321	0.000231086	yes	down
48	MT-CO1	-1.225122276	9.24E-12	yes	down
49	MECR	-1.202541622	0.000128716	yes	down
50	VDAC1	-1.169265467	2.39E-32	yes	down
51	MRPL12	-1.155762804	1.59E-08	yes	down
52	GTPBP3	-1.135444331	5.94E-25	yes	down
53	MRPS7	-1.126888236	7.63E-08	yes	down
54	TIMM10	-1.098781556	0.00000494	yes	down
55	MPV17L	-1.084770164	1.01E-25	yes	down
56	MTERF4	-1.056864167	6.99E-08	yes	down
57	METAP1D	-1.039330537	1.48E-10	yes	down
58	MICU3	-1.030208227	0.00000529	yes	down
59	MRPL28	-1.014762015	9.25E-68	yes	down
60	MRPL58	-0.994785295	1.98E-08	yes	down
61	TOMM40	-0.983363047	7.5E-27	yes	down
62	MRPS9	-0.982802349	0.000000113	yes	down
63	MTIF2	-0.979328483	0.00014144	yes	down
64	ATP5C1	-0.96085164	2.99E-10	yes	down
65	MRPS28	-0.956141579	5.16E-11	yes	down
66	TIMM8B	-0.942996203	3.09E-14	yes	down
67	MIPEP	-0.934205035	1.42E-44	yes	down
68	NARS2	-0.930898935	2.24E-10	yes	down
69	TIMM29	-0.922455134	6.94E-11	yes	down
70	MTERF2	-0.919517723	4.33E-25	yes	down
71	TIMM23	-0.914489775	2.62E-17	yes	down
72	SARS2	-0.91377152	1.62E-23	yes	down
73	ATP5O	-0.901383771	6.6E-09	yes	down
74	MRPL41	-0.88688871	1.53E-16	yes	down
75	MRPL40	-0.876291921	2.61E-09	yes	down
76	OPA3	-0.867723964	7.42E-13	yes	down
77	PCK2	-0.853377215	1.73E-18	yes	down
78	COX4I1	-0.827404934	7.76E-14	yes	down
79	CLUHP3	-0.821107481	4.33E-12	yes	down
80	PARS2	-0.672588922	0.000122244	yes	down
81	TOMM20	-0.661148943	5.27E-10	yes	down
82	IDH2	-0.616441349	2.12E-19	yes	down
83	AIFM2	-0.615068995	1.05E-41	yes	down

Table S1. Total down-regulated mitochondria-related genes upon CVB-D treatment in A549 cells was shown. The genes expression was compared between control and 30 μM CVB-D treated cells by transcriptome analysis. The mitochondria-related genes down-regulated upon CVB-D treatment was shown.

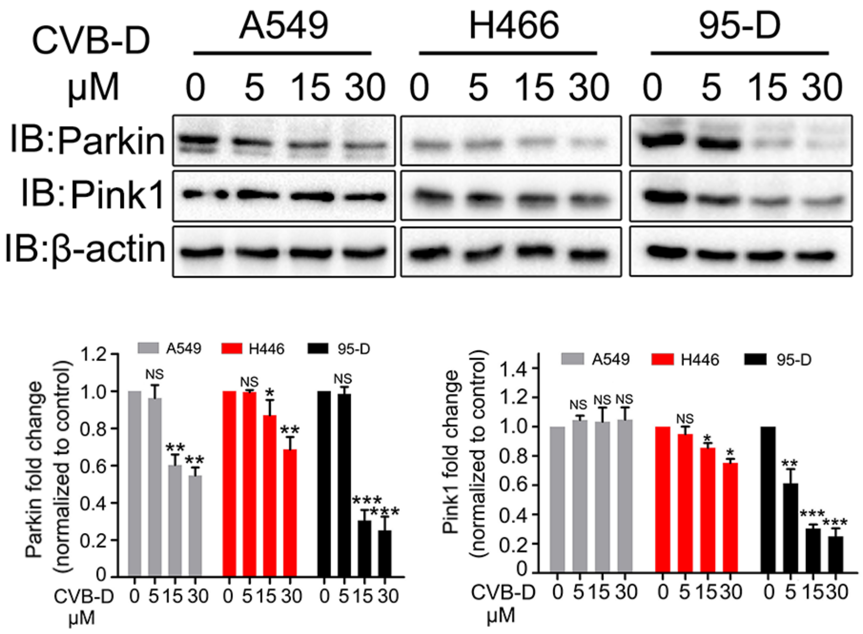

Figure S1. Immunoblotting analysis of Parkin and Pink1 expression upon CVB-D treatment in lung cancer cells. The expression of Parkin and Pink1 in lung cancer cells treated with or without 30 μM CVB-D for 24 h was detected by immunoblotting. Beta-actin was used as loading control.

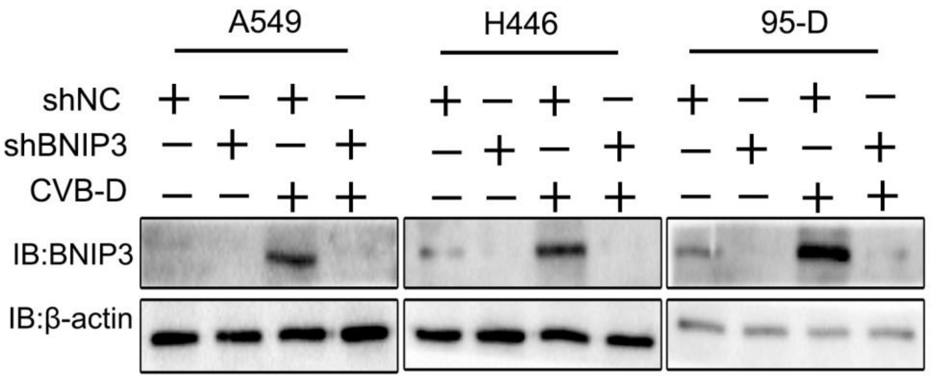

Figure S2. Immunoblotting analysis of BNIP3 expression upon CVB-D treatment in lung cancer cells. Western blotting analysis was performed to detect the expression of BNIP3 in shBNIP3 or shNC lung cancer cells treated with or without 30 μM CVB-D for 24 h. Beta-actin was used as loading control.

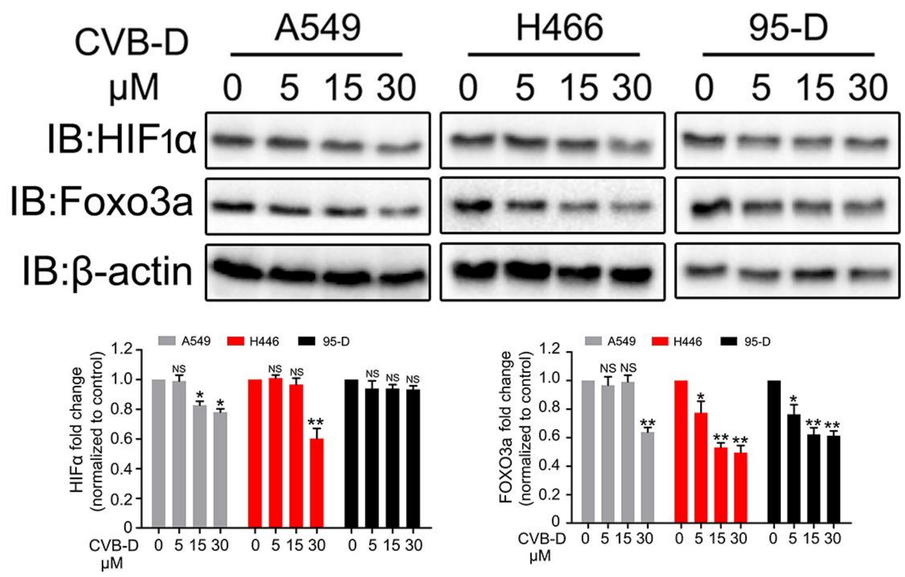

Figure S3. Immunoblotting analysis of HIF1α and FOXO3a expression upon CVB-D treatment in lung cancer cells. The expression of HIF1α and Foxo3a in lung cancer cells treated with or without 30 μM CVB-D for 24 h was detected by immunoblotting. Beta-actin was used as loading control.
